# Conserving evolutionarily distinct species is critical to safeguard human well-being

**DOI:** 10.1038/s41598-021-03616-x

**Published:** 2021-12-17

**Authors:** Rafael Molina-Venegas

**Affiliations:** Universidad de Alcalá, Department of Life Sciences, Global Change Ecology and Evolution Group, Alcalá de Henares, Madrid, Spain

**Keywords:** Ecology, Ecosystem services, Evolution, Phylogenetics

## Abstract

Although there is growing interest in safeguarding the Tree of Life to preserve the human benefits that are directly provided by biodiversity, their evolutionary distribution remains unknown, which has hampered our understanding of the potential of phylodiversity indicators to evince them. Here, I drew on a global review of plant benefits and comprehensive phylogenetic information to breakdown their evolutionary distribution and thereby show why the commonly used Phylogenetic Diversity and Evolutionary Distinctiveness indicators can unequivocally help to preserve these natural services. Beneficial species clumped within phylogenetically overdispersed genera and closely related species often contributed very few and redundant benefits, suggesting that multiple plant lineages are required to maintain a wide variety of services. Yet, a reduced number of species stood out as multi-beneficial and evolutionarily distinct plants relative to both the entire phylogeny and the subset of beneficial species, and they collectively contributed a higher-than-expected number of records for most types of benefits. In addition to providing a clear mechanistic understanding for the recently proved success of Phylogenetic Diversity in capturing plant benefits, these findings stress the decisive role that conservation programmes aimed at protecting evolutionarily distinct taxa will play in safeguarding the beneficial potential of biodiversity for the future.

## Introduction

Global conservation initiatives such as The Intergovernmental Science-Policy Platform on Biodiversity and Ecosystem Services are increasingly recognizing the importance of preserving the evolutionary heritage of biodiversity to safeguard Nature’s Contributions to People^1,2^, this is, the myriad of benefits contributed by biodiversity to the quality of life for humans^3^. There are two related indicators that allegedly interlink these natural benefits with the evolutionary history of species^4^, namely, the Phylogenetic Diversity (PD) and Evolutionary Distinctiveness (ED) metrics^5^. The PD indicator (minimum spanning path connecting a set of species in the phylogeny^6^) relies on the premise that distantly related taxa should provide, on average, different types of services, as they may show divergence in the functional traits that relate to the benefits^7^. Thus, the conviction is that by maximizing the retention of PD (hereafter ‘PD_max_’) we would maximize the retention of both, presently known benefits and possibly future ones that are yet to be discovered or documented^4^. Although this conservation phylogenetics perspective has fueled intense scientific debate^8–10^ and has long remained largely theoretical^11^, the framework has received some empirical support using plant genera as a case study^12,13^, hence bringing new promising insights for conservation practice^5^. On the other hand, the ED metric (a measure of how isolated a species is on the phylogeny^14^) has been adopted by global conservation initiatives such as the EDGE of Existence programme, which aims at preserving the world’s most evolutionarily distinct and endangered species^15,16^. The premise is that evolutionarily distinct species represent uniquely divergent genomes^17^ and hence putatively unique feature diversity to preserve for the future.

It is important to note that the success of phylodiversity indicators in capturing known biodiversity benefits will ultimately rely on the exact distribution of the latter in the phylogeny, a gap of knowledge that remains largely unexplored besides a few local accounts^18,19^. For example, the extent to which biodiversity benefits are prominently provided by the most evolutionarily distinct taxa, which are putative targets in ED-oriented conservation programmes^16^, has not been evaluated. Thus, a mechanistic and empirically supported understanding on why phylodiversity indicators can efficiently capture the beneficial potential of biodiversity is missing. Here, I drew on a global review of plant services (15,834 records sorted across 25 standard types of benefits^20^), comprehensive phylogenetic information, and analytical methods borrowed from the eco-phylogenetic literature to breakdown their evolutionary distribution and thereby show why the PD and ED indicators are empirically trustable means to safeguard known plant benefits beyond long argued theoretical expectations^11^.

## Results and discussion

**Figure 1:**
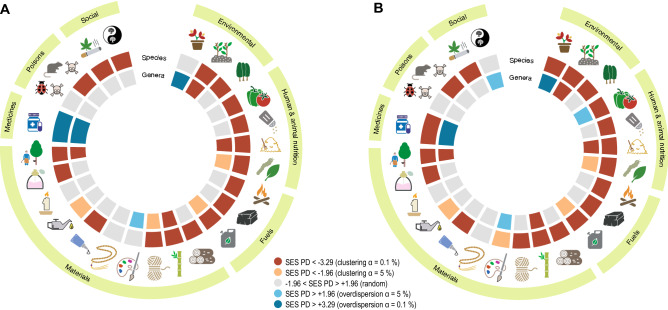
Types of plant benefits analyzed in the study. (**A**) and (**B**) show the results of the phylogenetic diversity (PD) analysis for the ‘full’ (all beneficial species) and ‘congeneric’ (a subset restricted to the species that showed at least one beneficial congeneric) datasets, respectively. The color of the sectors in the inner tracks represents the statistical significance of the PD tests (averaged SES PD scores, two-tailed tests) conducted for each type of benefit at the genus and species level, respectively. The exact averaged SES PD scores with 95% confidence intervals (representing phylogenetic uncertainty in SES score estimations) are provided in Supplementary Table 1, and they were considered significant for a given nominal alpha only if confidence intervals laid completely above (higher than expected) or below (lower than expected) the corresponding threshold (see legend). From twelve o’clock and clockwise: ornamental, soil improvers, hedges/shelters, human food, food additives, vertebrate food, invertebrate food, fuelwood, charcoal, biofuels, timber, cane, fibres, tannins/dyestuffs, beads, resins/gums, lipids, waxes, scents/essential oils, rubber, medicines, invertebrate poisons, vertebrate poisons, smoking materials/drugs and symbolism/inspiration.

**Figure 2:**
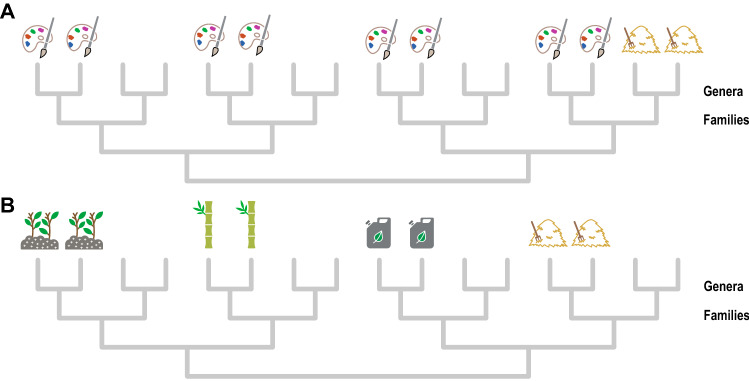
Hypothetic representation of plant benefits in the phylogeny. Phylogenetic nodes representing the same taxonomic rank (genus or family) are placed at the same height in the tree. (**A**) In this example, the benefit ‘dyestuffs’ is clumped in phylogenetically overdispersed genera belonging to four different families, while the benefit ‘fodder’ is uniquely provided by one genus. Assuming that the distribution of these benefits in the phylogeny were unknown and a conservation capacity limited to four species, a PD_max_ sampling strategy could be desirable for capturing species providing dyestuffs relative to an alternative strategy aimed at preserving, for example, one single family. This is because the PD_max_ regime will sample one species per family and thus a maximum of four dyestuff plants, whereas the family-restricted strategy will capture a maximum of two dyestuff plants. In contrast, the PD_max_ regime will always fail to capture the maximum possible number of fodder plants because only one fodder species could be sampled. (**B**) The plant benefits ‘soil improvers’, ‘cane’, ‘biofuel’ and ‘fodder’ are differentially provided by family clades (high phylogenetic turnover between the benefits), and thus the probability that PD_max_ scores four different benefits (*P* = 0.5^4^ = 0.0625) when sampling four species is almost one order of magnitude higher than that of getting the same result at random (*P* = 16/1820 = 0.0088).

The complete set of beneficial species (n = 9521, hereafter ‘full’ dataset) showed significantly high PD relative to the pool of seed plants [SES = 4.109 ± 0.058 (95% confidence interval)], indicating that beneficial species are widespread distributed across the phylogeny. However, the same analysis but restricted to the species that showed at least one beneficial congeneric (85.7% of the species in the full dataset, hereafter ‘congeneric’ dataset) resulted in a strong clustering pattern (SES = − 10.312 ± 0.0612), suggesting that genera represented by only one species in the full dataset vastly contributed to PD. On the other hand, beneficial genera (i.e. beneficial species collapsed into single tips representing genera) showed phylogenetic overdispersion (SES = 6.583 ± 0.031 and SES = 6.113 ± 0.017 for the full and congeneric datasets, respectively), revealing that most beneficial plants are highly packed in distantly related clusters of species across the entire phylogeny. Most individual types of benefits showed strong phylogenetic clustering at the species level regardless of the dataset except for medicinal plants, which were overdispersed and clustered for the full and congeneric datasets, respectively (Fig. 1). However, the genus-level phylogenetic structure of the benefits was complex and varied in opposite directions. For example, genera valuable as animal vertebrate and invertebrate food, biofuels (other than fuelwood and charcoal), cane material, rubber and soil improvers were significantly clustered in the phylogeny, while genera providing tannins/dyestuffs, medicinal and ornamental benefits showed the opposite pattern (Fig. 1). The genus-level clustering patterns reported here for some of the benefits indicate that a PD_max_ sampling regime, which aims at scoring disparate lineages^21^, may not efficiently capture them as they clump in a few sections of the phylogeny (Fig. 2a). For example, most animal vertebrate food is nowadays provided by Poaceae species as a result of long standing co-evolutionary dynamics with grazing mega-faunas^22^, and also by Fabaceae representatives likely due to their ability to fix atmospheric nitrogen and hence produce nutrient-rich tissues^19^. Accordingly, a recent study reported a poor performance of the PD_max_ strategy in capturing fodder plants as well as a few other genus-level phylogenetically clustered benefits such as biofuels and cane materials^13^. In contrast, the PD_max_ regime will more efficiently capture plant benefits that are packed in overdispersed genera (e.g. medicines, ornamental, human food and tannins/dyestuffs; Fig. 1), as PD_max_ will tend to more likely score such distantly related lineages (Fig. 2a). This would explain why the benefits ‘medicines’ and ‘human food’, two well-recognized Nature’s contributions to people^3^ that much fit to this phylogenetic scheme (Fig. 1), were successfully captured in the two studies that have so far tested the PD_max_ sampling strategy locally^12^ and across biogeographic realms^13^.

Although alpha phylogenetic diversity patterns inform on the ability of PD_max_ to capture each type of benefit separately, understanding its potential to retrieve subsets of species with complementary services requires quantifying the degree of specificity in the relationship between evolutionary lineages and benefits—i.e. the extent to which closely related taxa tend to supply the same benefit (Fig. 2b). Thus, in order to elucidate the extent to which phylogenetic clades differentially provide plant benefits, I explored phylogenetic beta diversity patterns (pβ_sor_) between the latter treating them as if they were “sites”^19,23^. Roughly, and providing that pβ_sor_ between types of benefits is high and mainly due to its “true” turnover component (pβ_sim_), higher than expected pβ_sim_ would indicate high specificity in the benefit-clade relationship (closely related taxa tending to supply the same benefit), whereas the opposite pattern would indicate low specificity instead (see Supplementary Fig. 1). I found that pβ_sor_ among types of benefits was very high (multiple-site pβ_sor_ = 0.94 ± 1.11e^−5^ and 0.94 ± 1.37e^−5^ for the full and congeneric datasets, respectively) and mostly due the pβ_sim_ component (multiple-site pβ_sim_ = 0.79 ± 5.45e^−5^ and 0.79 ± 3.36e^−5^, respectively). Strikingly, pβ_sim_ was significantly high in 91% and 89.67% of the pairwise comparisons (n = 300) in the full and congeneric datasets, respectively, and no comparisons showed lower than expected pβ_sim_ values (Supplementary Figs. 2–3 and Supplementary Table 1). At the genus level, pβ_sor_ was also high (multiple-site pβ_sor_ = 0.92 ± 3.55e^−6^ and 0.92 ± 5.40e^−6^ for the full and congeneric datasets, respectively) and mostly due to pβ_sim_ (multiple-site pβ_sim_ = 0.76 ± 2.20e^−5^ and 0.74 ± 1.84e^−5^, respectively), although the proportion of significantly high pβ_sim_ pairwise comparisons decreased (59.33% and 48.67%, respectively). Yet only one pβ_sim_ pairwise comparison was lower than expected in either genus-level dataset (Supplementary Figs. 4–5 and Supplementary Table 1). These figures reveal a deeply rooted turnover of plant services across the phylogeny that would explain why a PD_max_ sampling strategy can capture not only a greater-than-expected number of total benefits but more equitable distributions among the different types^13^.

**Figure 3:**
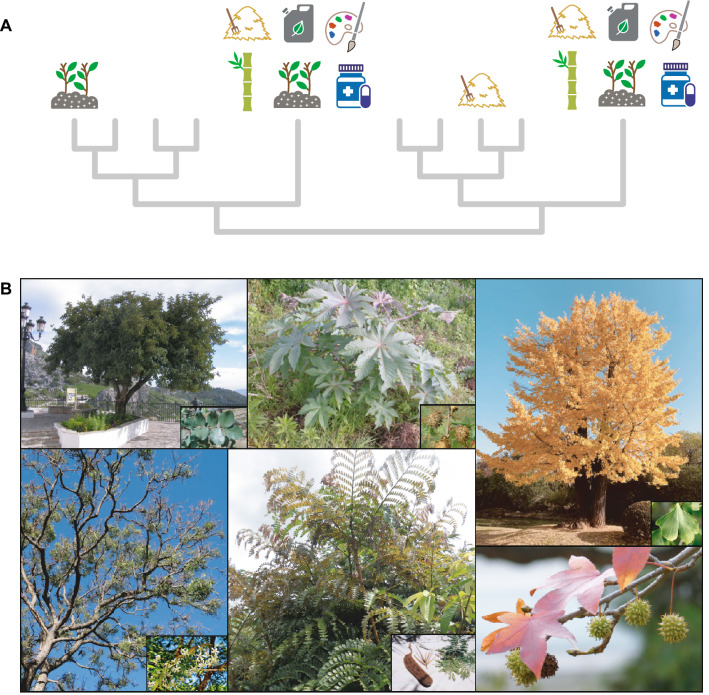
(**A**) Hypothetic representation of evolutionarily distinct and multi-beneficial species in the phylogeny (long terminal branches), a pervasive pattern that largely explains the success of the Phylogenetic Diversity metric in capturing plant benefits. (**B**) A selection of extremely evolutionarily distinct and multi-beneficial species (plants whose averaged evolutionarily distinctiveness values across the 1000 phylogenies analyzed were above the 97.5th percentile and contributed seven or more types of benefits). From left to right and up to bottom: *Ceratonia siliqua* (Fabaceae) and detail of the leaf (inset photo courtesy by José León), *Ricinus communis* (Euphorbiaceae) and detail of mature fruits (Photo by Scamperdale under CC-BY-NC license: https://www.flickr.com/photos/36517976@N06/3426117042/, inset courtesy by José León), *Ginkgo biloba* (Ginkgoaceae) and detail of the leaf (Photo by Alvan Nee), *Azadirachta indica* (Meliaceae) and flowers, *Pentaclethra macrophylla* (Fabaceae) with leaves, fruit and inflorescence (Photo and inset by Scamperdale under CC-BY-NC license: https://www.flickr.com/photos/36517976@N06/5646071190), and leaves and inflorescences of *Liquidambar styraciflua* (Altingiaceae).

In line with these phylogenetic patterns, compositional turnover (β_sim_) in plant benefits within genera and families (turnover in benefits between congeneric and confamiliar species, see Supplementary Fig. 6) was significantly low and particularly for large genera and families (Supplementary Figs. 7–8), which indicates that, overall, congenerics and confamiliars contributed very few and redundant benefits (83.74% of the species in the dataset provided just one or two benefits). In contrast, compositional nestedness (β_nes_) among congenerics and confamiliars was high in many cases (Supplementary Figs. 7–8), thus revealing the existence of a reduced number of plants that stood out as multi-beneficial species within their genus- and family-clades (Supplementary Fig. 6). Further, the median evolutionary distinctiveness of these multi-beneficial plants (subsets of beneficial species that contributed at least three, four, five, six, seven and eight types of benefits, respectively) was significantly high relative to both the entire phylogeny and the subset of beneficial species analyzed in the study (Supplementary Table 2). Furthermore, multi-beneficial plants collectively contributed a higher-than-expected number of records for most types of benefits and particularly for fuels (fuelwood, charcoal, and biofuels) and some materials such as lipids (Supplementary Tables 3–4), and this was true even for some of the rarest benefits. For example, 22 out of 35 species that were valuable as biofuels (the second rarest benefit, Supplementary Table 1) contributed at least four additional types of benefits while representing only 3.2% of the species in the dataset (Supplementary Table 3). This result highlights the functional uniqueness of the few multi-beneficial and evolutionarily distinct species evinced in the study (Fig. 3), a minority of plants that encapsulate a great amount of evolutionary history and will, therefore, often be selected by a PD_max_ sampling regime^24^. This finding stresses the decisive role that conservation programmes aimed at protecting evolutionarily distinct and endangered taxa, such as the EDGE of Existence programme^15,16^, will play in safeguarding a wide variety of known benefits for the future. For example, the maidenhair tree (*Ginkgo biloba*), the most evolutionarily distinct seed plant in existence and a multi-beneficial plant (7 types of benefits according to the data), is catalogued as Endangered^25^. Nonetheless, the ecological apparency hypothesis predicts human preference for readily available widespread species^26^, suggesting that, hopefully, the conservation status of many multi-beneficial and evolutionarily distinct species may be of least concern. However, this hypothesis is yet to be evaluated, and the fact is that the conservation status of most seed plants remains unknown^27^. Beyond assessing species’ extinction risk, future studies might help to elucidate whether evolutionarily distinct and multi-beneficial species can be featured by means of specific combinations of functional and/or life-history traits.

Following our failure to achieve the 20 Aichi biodiversity targets^28^, nations are now working together to agree the post-2020 Global Biodiversity Framework (GBF), an ambitious initiative aimed at guaranteeing a healthy biodiversity and sustainable development with a 2050 horizon. Importantly, recognizing all the benefits or services on people that are directly provided by biodiversity is at the heart of the post-2020 GBF. Thus, by providing a mechanistic and empirically supported understanding on why PD and ED indicators can efficiently capture known plant benefits, this study factually positions phylodiversity as a powerful means for achieving some of the ambitious environmental goals that humanity must face in the coming decades^2^. For example, knowing that ethnobotanical knowledge is vastly under-documented^29^, efforts should be directed to prospect taxa that, while showing little or no apparent medicinal properties, a PD_max_ sampling regime could evince as medically valuable, and special attention should be paid to evolutionarily distinct taxa with unknown benefits. The instrumental motivation for preserving plant biodiversity this study speaks most directly can counter beliefs that biodiversity should be viewed as intrinsically valuable^30^. Yet, a transversal principle across such somewhat irreconcilable worldviews is that highly biodiverse ecosystems are desirable over depauperated ones, and there is an increasing number of people showing preference for complexity and distinctiveness. Evolutionary history can inform these properties of biodiversity^31^, which bodes well for the promising discipline of conservation phylogenetics. My hope is that this study serves to reinforce our commitment to safeguarding the Tree of Life and hence the beneficial potential of biodiversity for the future.

## Methods

### Dataset of beneficial plants

I collated a species-level dataset of plant benefits (presence/absence data) starting from the information gathered by Kleunen et al.^32^. These authors extracted data from the WEP database (National Plant Germplasm System GRIN-GLOBAL; https://npgsweb.ars-grin.gov/gringlobal/taxon/taxonomysearcheco.aspx, Accessed 7 Jan 2016), which is based on the book by Wiersema and León^20^. Their dataset included 84 categories and subcategories of plant benefits pertaining human and animal nutrition, materials, fuels, medicine, useful poisons, social and environmental benefits. Subcategories of benefits, which often included very few records, were merged here into 25 standard and major categories following the guidelines in the Economic Botany Data Collection Standard^33^ as in Molina-Venegas et al.^13^, namely ornamental plants, soil improvers, hedging/shelter, human food, human-food additives, vertebrate food, invertebrate food, fuelwood, charcoal, other biofuels, timber, cane/stems, fibres, tannins/dyestuffs, beads, gums/resins, lipids, waxes, essential oils/scents, latex/rubber, medicines, invertebrate poison, vertebrate poison, smoking materials/drugs and symbolic/inspirational plants (Fig. 1). A few records (n = 93) that could not be assigned to any of the above categories were disregarded, and so was the category ‘gene source’ because unlike other benefits, any species is intrinsically a potential gene donor and hence there is not a clear link between the benefit and species features. Note that this is not to say that preserving genetic diversity, which indeed is the underlying message of this research, is a meaningless goal. Infraspecific taxa were collapsed at the species level, and the very few fern taxa in the original database^32^ were excluded. In total, I gathered 15,834 plant-benefit records sorted in a matrix of 25 types of benefits and 9521 species of seed plants. Most species (83.74%) provided only one or two benefits representing 62.83% of the records in the dataset, and the maximum number of benefits per species was 10 (only three species). Although the WEP database is the largest species-level database on plant benefits^32^, it does not claim to be comprehensive^20^. Yet, the size of the dataset I gathered here represented 76.19% of the total seed-plant genus-level records collated for the same types of benefits in a more comprehensive survey by Molina-Venegas et al.^13^ that based on *Mabberley’s Plant-book*^34^. Moreover, the total number of records per category (at the genus-level) strongly correlated between the datasets (Pearson r = 0.94, *p* < 0.001) and so did the standardized genus-level phylogenetic diversity (averaged SES PD scores) of the categories (Pearson r = 0.81, *p* < 0.001). These figures suggest that, while still suffering from our limited knowledge on plant benefits^29^, the species-level dataset analyzed here represents a reasonable and unbiased sample of the global seed-plant beneficial feature diversity.

### Phylogenetic information

Phylogenetic information on seed plants is incomplete. As such, even the most comprehensive and sophisticated molecular phylogeny published hitherto^35^ only accounts for ~ 23% of all accepted seed plant species (~ 322,000 according to *Plants of the World Online* portal of Kew Sciences; http://www.plantsoftheworldonline.org and ~ 330,000 according to a very recent account^36^). Further, 28% of all accepted genera of seed plants are missing from this phylogeny^13^. Nevertheless, although we are still far from achieving a comprehensive species-level phylogeny for seed plants, phylogenetic uncertainty can operatively be accommodated in the analyses^37^. Rather than analyzing one single incomplete phylogeny, a distribution of possible trees can be rendered using a systematic procedure to randomize phylogenetically uncertain taxa in the clades that most certainly contain them (using taxonomically informed and educated decisions^36^). Then, confidence intervals can be computed for the target metrics so that the impact of phylogenetic uncertainty in the analyses can be estimated^13,38^.

In order to draw a distribution of possible species-level seed plant phylogenies, I started from the exact set of 100 genus-level trees (after removing pteridophytes) that were assembled in a previous global study by Molina-Venegas et al.^13^. These genus-level time-calibrated phylogenies were constructed based on the GBOTB tree^35^, which included phylogenetic information for 72% of all accepted seed plant genera (9505 out of 13,202). Thus, the missing genera were randomized in the tree following the workflow proposed by Rangel et al.^37^ to generate 100 complete genus-level trees (see Molina-Venegas et al.^13^ for full details on this procedure). I retrieved the total number of accepted species per genus from *Plants of the World Online* and labelled them using an alpha-numerical code. For example, the 49 accepted species in the genus *Abies* were labelled as *Abies-*1, *Abies-*2, *Abies-*3, …, *Abies-*49. Then, I derived 100 stochastic species-level trees from each genus-level phylogeny by randomly resolving infrageneric relationships among the retrieved species using a pure-birth model of evolution^39^. This procedure rendered a distribution of 100 species-level seed plant phylogenies (321,817 tips) per genus-level tree, making a total of 10,000 possible phylogenies. Because the identity of the beneficial species is missing in the so-generated phylogenies, I assigned an identity to each beneficial species in the dataset at random, and this labelling correspondence was maintained across the 10,000 trees. For example, the beneficial species *Abies cephalonica* and *Abies pinsapo* were respectively represented by *Abies-*4 and *Abies-*17 in the trees (note that their phylogenetic placement below the *Abies* crown node was simulated using a pure-birth model of evolution and thus differed across the trees). After verifying that species-level phylogenetic uncertainty had a negligible effect in the analyses (Supplementary Fig. 9), I randomly picked 10 trees from each individual distribution of species-level phylogenies (100 different distributions, one per genus-level tree) and used them for the analyses. Thus, all the species-level phylogenetic analyses described below were conducted and results averaged across 1000 different phylogenies and genus-level analyses were carried out across 100 trees. Note that for practical reasons the species-level phylogenies used here do not incorporate available infrageneric topological information in the GBOTB tree. To circumvent this putative limitation (because we can hardly be certain that available infrageneric topological information in the GBOTB tree represents the “true” evolutionary relationships), I only considered SES scores as significant for a given nominal alpha if 95% confidence intervals (representing phylogenetic uncertainty in SES score estimations) laid completely above (higher than expected) or below (lower than expected) the corresponding threshold.

### Phylogenetic alpha diversity

Investigating phylodiversity patterns across different phylogenetic scales can help to achieve new and more complete insights into the evolutionary distribution of feature diversity^40^. Thus, for each phylogeny analyzed, I computed the amount of evolutionary history (PD) that was encapsulated by all beneficial taxa as a whole and by each subset of taxa contributing the same benefit at two different phylogenetic grains, namely genus and species level. To create a matrix of plant benefits at the genus level, I simply collapsed congenerics records into individual observations for each type of benefit. Because PD is not statistically independent of taxa richness and the former differed greatly between the types of benefits (Supplementary Table 1), I computed SES scores to make PD values comparable between them as:

1 $$\mathrm{SES}= \frac{{M}_{obs} - {M}_{null}}{{SD}_{null}}$$

where SES is the standardized effect size score for a given set of beneficial taxa, phylogeny and phylogenetic grain, M_obs_ is the observed PD value for the set, M_null_ is the mean of a null distribution of PD values generated by randomly drawing from the phylogeny the same number of taxa as in the focal set 999 times, and SD_null_ is the standard deviation of the null distribution^41^. SES scores were averaged across 100 and 1000 phylogenetic hypotheses in the genus- and species-level analyses, respectively, and 95% confidence intervals were computed in each case. To evaluate the impact of beneficial species that were the only representatives of their corresponding genera (14.3% of the species in the dataset), I conducted all the phylogenetic analyses of the study using (i) all beneficial species (‘full’ dataset, n = 9521 species) and (ii) a subset of the latter where singleton beneficial genera were excluded (‘congeneric’ dataset; n = 8163 species).

### Phylogenetic beta diversity

I characterized phylogenetic beta diversity patterns among types of benefits (phylogenetic dissimilarity) using the PhyloSor index^42^. The PhyloSor metric represents the proportion of evolutionary units (typically branch-length) that is shared between two samples (here types of benefits), and it ranges between 0 (no branch-length is shared) and 1 (all branch-length is shared). Thus, phylogenetic beta diversity (pβ_sor_) is defined as 1 – PhyloSor index^43^. The pβ_sor_ metric can be decomposed into two additive components, namely “true” phylogenetic turnover (pβ_sim_) and nestedness (pβ_nes_)^23^. While pβ_nes_ is the fraction of PBD that emerges due to differences in PD between the samples, the pβ_sim_ component implies the replacement of an exact amount of branch-length, the branch-length that is replaced being unique to each sample. In words, pβ_sim_ represents the phylogenetic dissimilarity between samples after accounting for differences in PD, and it provides insight on the phylogenetic depth at which turnover of lineages between samples occurs if analyzed in a null model context^23^. As such, the observed pβ_sim_ can be compared against a null distribution of pβ_sim_ values generated by shuffling taxa labels across the tips of the phylogeny representing beneficial taxa (so that compositional dissimilarity between samples remains unchanged but phylogenetic distances are shuffled) and a SES score can be computed (Eq. 1). Significantly low SES pβ_sim_ would indicate that replacement of lineages between the samples tends to occur towards the tips of the phylogeny (lower than expected pβsim for the given compositional dissimilarity), whereas significantly high SES pβ_sim_ would indicate that replacement involves deeper phylogenetic nodes^19,44^. Therefore, lower than expected SES pβ_sim_ between two types of benefits would indicate low specificity between phylogenetic clades and benefits (i.e. closely related taxa tend to provide different benefits), and higher than expected values would indicate high specificity in this relationship (i.e. closely related taxa tend to provide the same benefit) (see Supplementary Fig. 1). Here, I computed pairwise pβ_sim_ values between each pair of benefit types and the corresponding SES scores using Eq. 1 and the null model described above (i.e. taxa shuffling across beneficial taxa 999 times). SES scores were averaged across 100 and 1000 phylogenetic hypotheses in the genus- and species-level analyses, respectively, and 95% confidence intervals were computed in each case. To get an idea of the overall phylogenetic dissimilarity and turnover among all types of benefits, I also computed multi-site pβ_sor_ and pβ_sim_ values^43^.

### Differentiation in contributed benefits among congenerics and confamiliars

To complement the analyses described above, I further explored whether congeneric and confamiliar species provided different types of services. To do so, I computed multiple-site dissimilarities in benefits among congenerics and confamiliars (multiple-site β_sor_ and its additive components β_sim_ and β_nes_^45^) using the Sorensen index (1 - Sorensen), treating species as if they were “sites” and benefits as “species” (see Supplementary Fig. 6). For a given genus or family, multiple-site β_sor_ would be equal to 0 if all congenerics or confamiliars provide the exact same types of benefits (maximum redundancy), and otherwise β_sor_ would be greater than 0 and up to 1 (minimum redundancy). Significantly high β_sim_ values would indicate high complementarity between congenerics or confamiliars in terms of beneficial value, whereas significantly high β_nes_ would indicate strong differences in the number of contributed benefits and therefore the presence of species that stand out as multi-beneficial plants among their congenerics or confamiliars (see Supplementary Fig. 6). The observed multiple-site β_sor_, β_sim_ and β_nes_ values of each beneficial genus and family in the dataset were compared against null distributions generated by randomly drawing from the pool of beneficial species the same number of species as in the target genus or family 999 times. However, the null distributions were odd and did not fit normality (particularly for small-sized genera, Supplementary Fig. 10), which prevented from using SES scores. Instead, I calculated non-parametric ES values based on the probability *P* for the observed β_sor_, β_sim_ and β_nes_ values to be higher than expected given the corresponding null distributions as:

2 $$P= \frac{number \left(null<obs\right)+ \frac{number \left(null=obs\right)}{2}}{1000}$$

then subtracting 0.5 to *P* and multiplying the result by 2 to obtain ES scores^46,47^. ES scores vary between − 1 and 1, with values close to − 1 and 1 indicating that the observed β_sor_, β_sim_ and β_nes_ are lower and higher than expected based on the null distributions, respectively. Beneficial genera and families represented by one single species in the dataset were not considered for this analysis because at least two “sites” are required to compute beta diversity metrics.

### Evolutionary distinctiveness of multi-beneficial species

Firstly, I computed the evolutionary distinctiveness (ED) of each seed plant species (n = 321,817) using the fair proportion approach^15^. Then, I used this data to test whether the median ED of multi-beneficial species, this is, those that respectively provided at least three (n = 1548), four (n = 666), five (n = 302), six (n = 143), seven (n = 73) and eight (n = 39) types of benefits, was significantly low or high relative to (i) the entire phylogeny and (ii) the subset of beneficial species analyzed in the study. To do so, I compared the median ED of each subset of multi-beneficial species against random distributions of median ED values generated by randomly drawing the same number of species from the entire phylogeny and the set of beneficial species 999 times, respectively (SES scores, Eq. 1). The median was used as a metric of central tendency instead of the arithmetic mean because ED values were strongly skewed by a small proportion of extremely large ones and thus the median provided a better representation of their central tendency. SES scores were averaged across 1000 species-level phylogenetic trees and 95% confidence intervals were computed in each case.

To elucidate if multi-beneficial plants contributed a higher-than-expected number of records of each type of benefit, I tested the null hypothesis that the species in each multi-beneficial subset provided, as a whole, a number of benefits of each type in direct proportion to their representation in the pool of beneficial species (Chi-square tests with one degree of freedom). For example, the subset of multi-beneficial plants contributing three or more benefits represented 16.3% of the total pool of beneficial species, and thus the null expectation is that they will contribute 16.3% of the records of each type of benefit. All the analyses were conducted in R v. 4.0.3^48^ using the packages *picante*^49^, *phytools*^39^, *betapart*^43^ and *phyloregion*^50^.

## Supplementary Information

Supplementary Information.

Supplementary Table S1.

## Data Availability

The dataset of beneficial plants is available in figshare repository (https://doi.org/10.6084/m9.figshare.16877122).

## Code availability

All the code used in this research is available as functions that were implemented in published R packages.
