# Supplementary material for: Conserving evolutionarily distinct species is critical to safeguard human well-being
Source: Sci Rep. 2021 Dec 17;11:24187. doi: 10.1038/s41598-021-03616-x (PMC8683420; doi:10.1038/s41598-021-03616-x)
Supplement: Supplementary file 1 — Supplementary Information. [file 41598_2021_3616_MOESM1_ESM.pdf]

Conserving evolutionarily distinct species is critical to safeguard human well-being

Rafael Molina-Venegas

Correspondence to: rafmolven@gmail.com

Content

Supplementary Figures 1–10.

Legend for Supplementary Table 1.

Supplementary Tables 2–4.

Table S1. Averaged SES scores with 95% confidence intervals for the phylogenetic diversity (PD) that is encapsulated by each plant benefit (full and congeneric datasets at the species and genus levels), pairwise phylogenetic beta diversity ($p\beta_{sim}$ component) between plant benefits (full and congeneric datasets at the species and genus levels), and ES scores of multiple-site compositional beta diversity (β_{sor}) and its turnover (β_{sim}) nestedness (β_{nes}) components in plant benefits among congenerics and confamiliars.

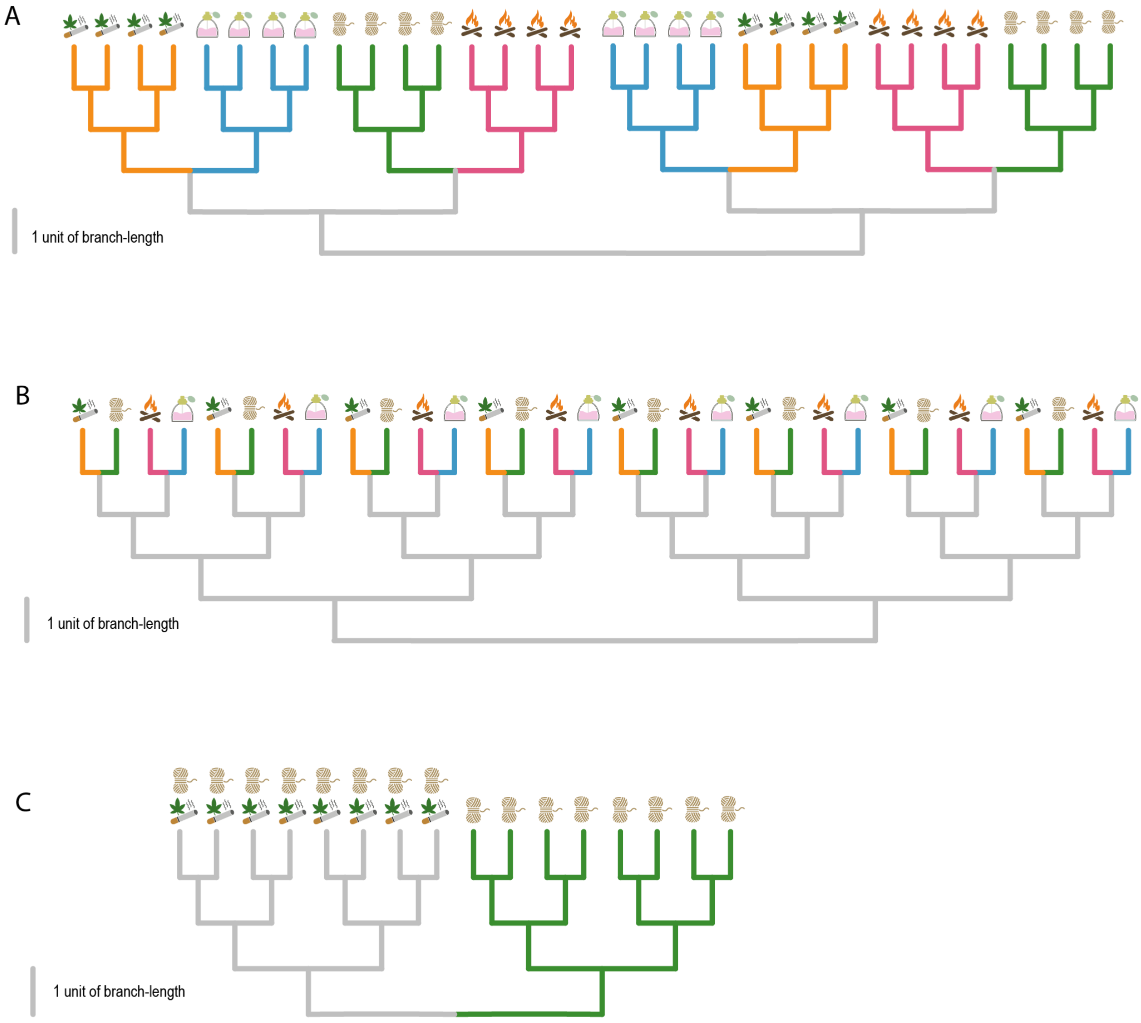

Supplementary Figure 1. Conceptual framework for the use of phylogenetic beta diversity ($p\beta_{sor}$) as an indicator of the degree of specificity in the relationship between phylogenetic clades and plant benefits. The $p\beta_{sor}$ metric has been traditionally used to measure phylogenetic dissimilarity between sites or communities. Here, however, I treated types of benefits as if they were "sites" instead to estimate the degree of specificity in the relationship between phylogenetic clades and plant benefits. By analogy, taxa sharing the same type of benefit "co-occur" in a "site", and those providing more than one type of benefit "occur" in more than one "site". (A) Each taxon represented in the phylogeny (tips) provides only one type of benefit (smoking materials, fibres, fuelwood, or scents). The phylogenetic branches in orange, blue, green, and magenta are uniquely represented by each type of benefit, whereas grey branches are shared by at least two benefits. Compositional dissimilarity between any pair of benefits is maximum because

there are no shared taxa between them, and phylogenetic dissimilarity ($p\beta_{sor}$) is also very high because the benefits share very few branches (only the deepest ones). Further, because each of the benefits encapsulates an identical amount of phylogenetic diversity (PD, 18 units of branch length each), $p\beta_{sor}$ is entirely due to "true" phylogenetic turnover ($p\beta_{sor} = p\beta_{sim}$). The phylogenetic turnover between any two types of benefits can be expressed as the averaged phylogenetic depth that is required to connect all possible pairs of taxa of different type. In this example, connecting such taxa requires traversing deep phylogenetic nodes in all cases. As such, if taxon identity would be shuffled across the phylogenetic tips to compute null $p\beta_{sim}$ values, the latter will most certainly be lower than the observed $p\beta_{sim}$ because the amount of shared branch length between taxa of different types will increase after the randomizations (as in figure B). Thus, higher than expected $p\beta_{sim}$ relative to the null model "taxa shuffling" indicate a high specificity in the relationship between phylogenetic clades and the benefits. Note that compositional dissimilarity between the benefits (i.e. the extent to which they share taxa) is unaffected by this null model and remains maximum after shuffling species identity. (B) Compositional dissimilarity is also maximum (no shared taxa between the benefits). However, despite $p\beta_{sor}$ is still entirely due to the $p\beta_{sim}$ component (PD is equal between the benefits; 30 units of branch length each), now the benefits share a great amount of branch length (the phylogenetic depth that is required to connect pairs of taxa of different type is shallow). As such, if taxon identity would be shuffled across the phylogenetic tips to compute null $p\beta_{sim}$ values, the latter will most certainly be higher than the observed value, hence indicating a low specificity in the relationship between phylogenetic clades and the benefits (sister taxa provide different types of benefits). (C) Compositional dissimilarity is intermediate because half of the taxa are shared between the two benefits (i.e. half of the taxa provide both benefits). Now, $p\beta_{sor}$ is entirely due to phylogenetic nestedness ($p\beta_{sor} = p\beta_{nes}$) as the former emerges simply because the benefits differ in their PD value (30 and 15 units of branch length for fibres and smoking materials, respectively) and there is not branch length uniquely represented by smoking materials. Note that although $p\beta_{nes}$ will most certainly be higher than expected relative to the null model described above (because taxa shuffling will equalize PD values between the benefits), we may not conclude that there is a high specificity between phylogenetic clades and benefits; only the $p\beta_{sim}$ component of $p\beta_{sor}$ speaks on the strength of this relationship.

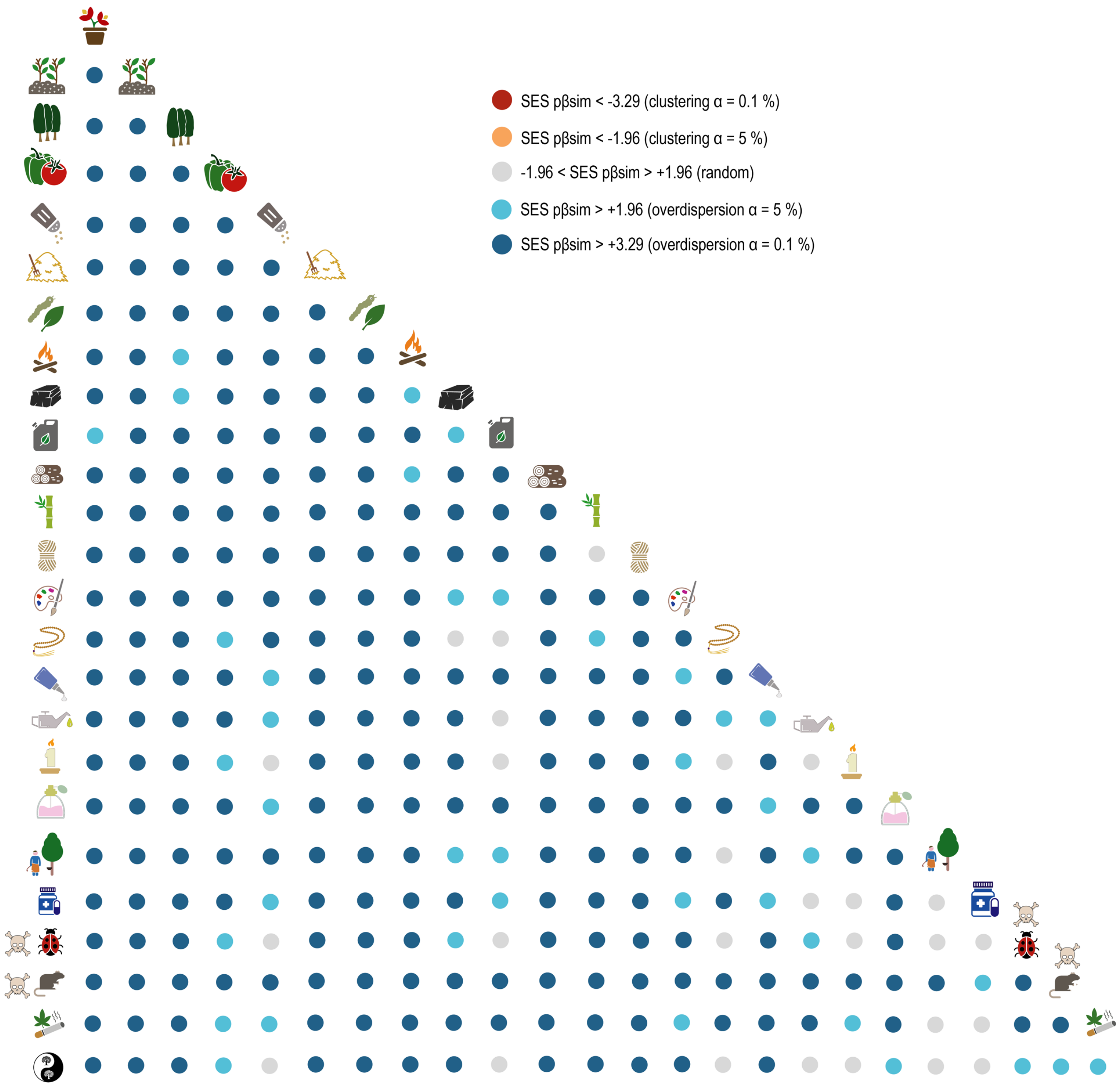

Supplementary Figure 2. Statistical significance (averaged Standardized Effect Size scores, two-tailed tests) of the "true" turnover component of phylogenetic beta diversity (SES $p\beta_{sim}$) between pairwise types of benefits for 5% and 0.1% nominal alpha (species level and full dataset). The exact averaged SES $p\beta_{sim}$ scores with 95% confidence intervals are provided in Supplementary Table 1, and they were considered significant only if 95% confidence intervals (representing phylogenetic uncertainty in SES score estimations) laid completely above (higher than expected) or below (lower than expected) the corresponding threshold (see legend).

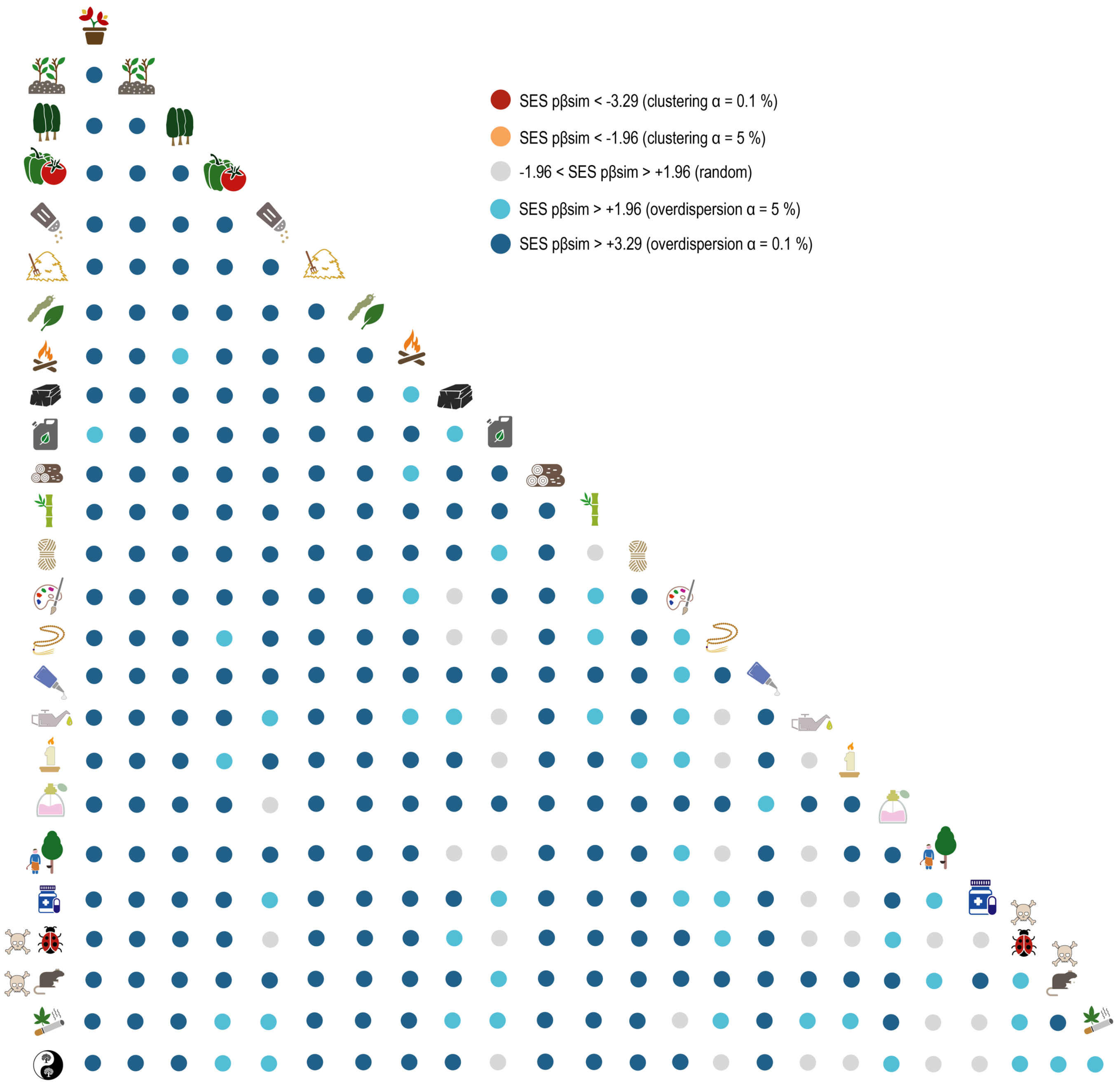

Supplementary Figure 3. Statistical significance (averaged Standardized Effect Size scores, two-tailed tests) of the “true” turnover component of phylogenetic beta diversity (SES $p\beta_{sim}$) between pairwise types of benefits for 5% and 0.1% nominal alpha (species level and congeneric dataset). The exact averaged SES $p\beta_{sim}$ scores with 95% confidence intervals are provided in Supplementary Table 1, and they were considered significant only if 95% confidence intervals (representing phylogenetic uncertainty in SES score estimations) laid completely above (higher than expected) or below (lower than expected) the corresponding threshold (see legend).

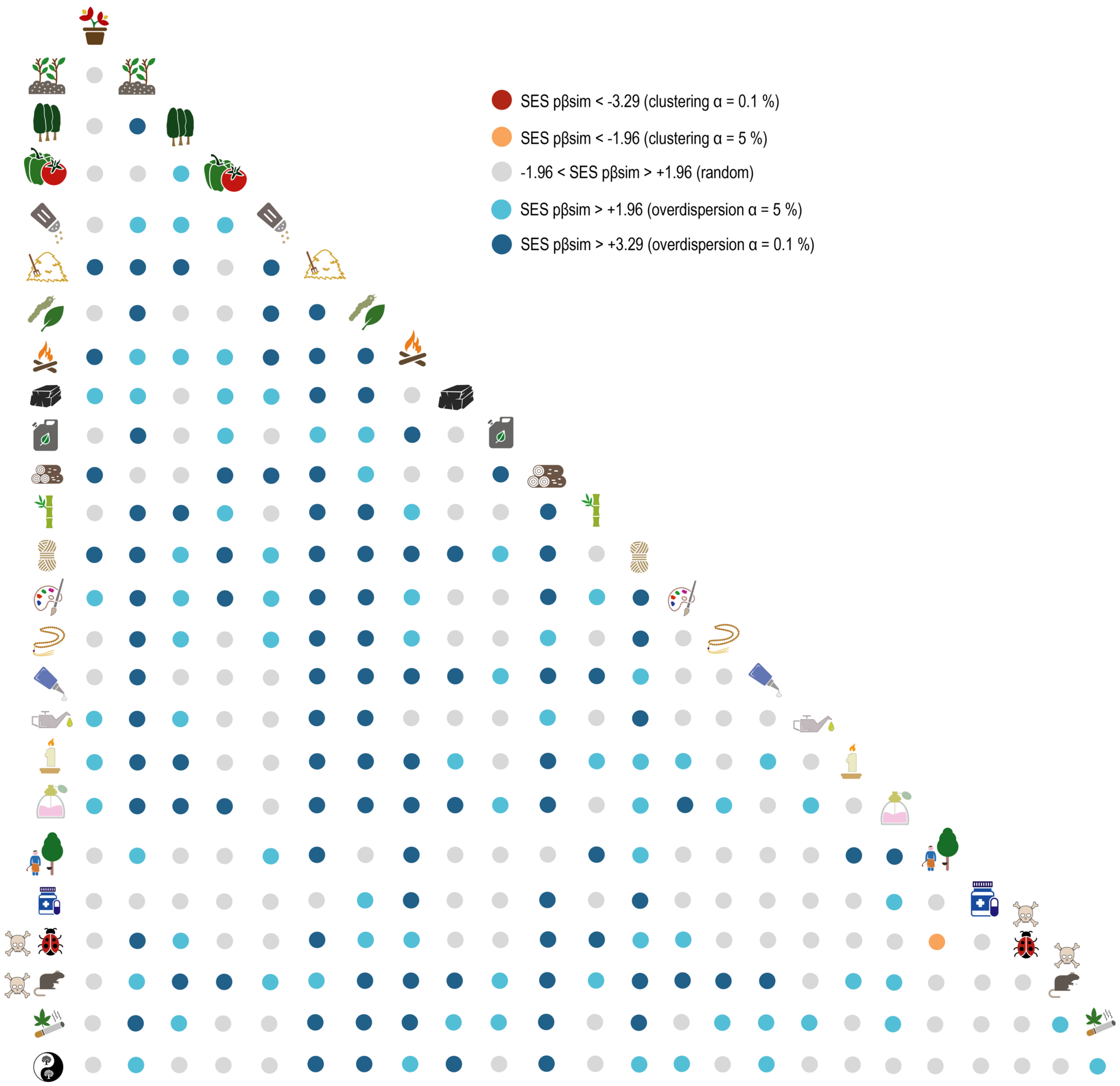

Supplementary Figure 4. Statistical significance (averaged Standardized Effect Size scores, two-tailed tests) of the "true" turnover component of phylogenetic beta diversity (SES $p\beta_{sim}$) between pairwise types of benefits for 5% and 0.1% nominal alpha (genus level and full dataset). The exact averaged SES $p\beta_{sim}$ scores with 95% confidence intervals are provided in Supplementary Table 1, and they were considered significant only if 95% confidence intervals (representing phylogenetic uncertainty in SES score estimations) laid completely above (higher than expected) or below (lower than expected) the corresponding threshold (see legend).

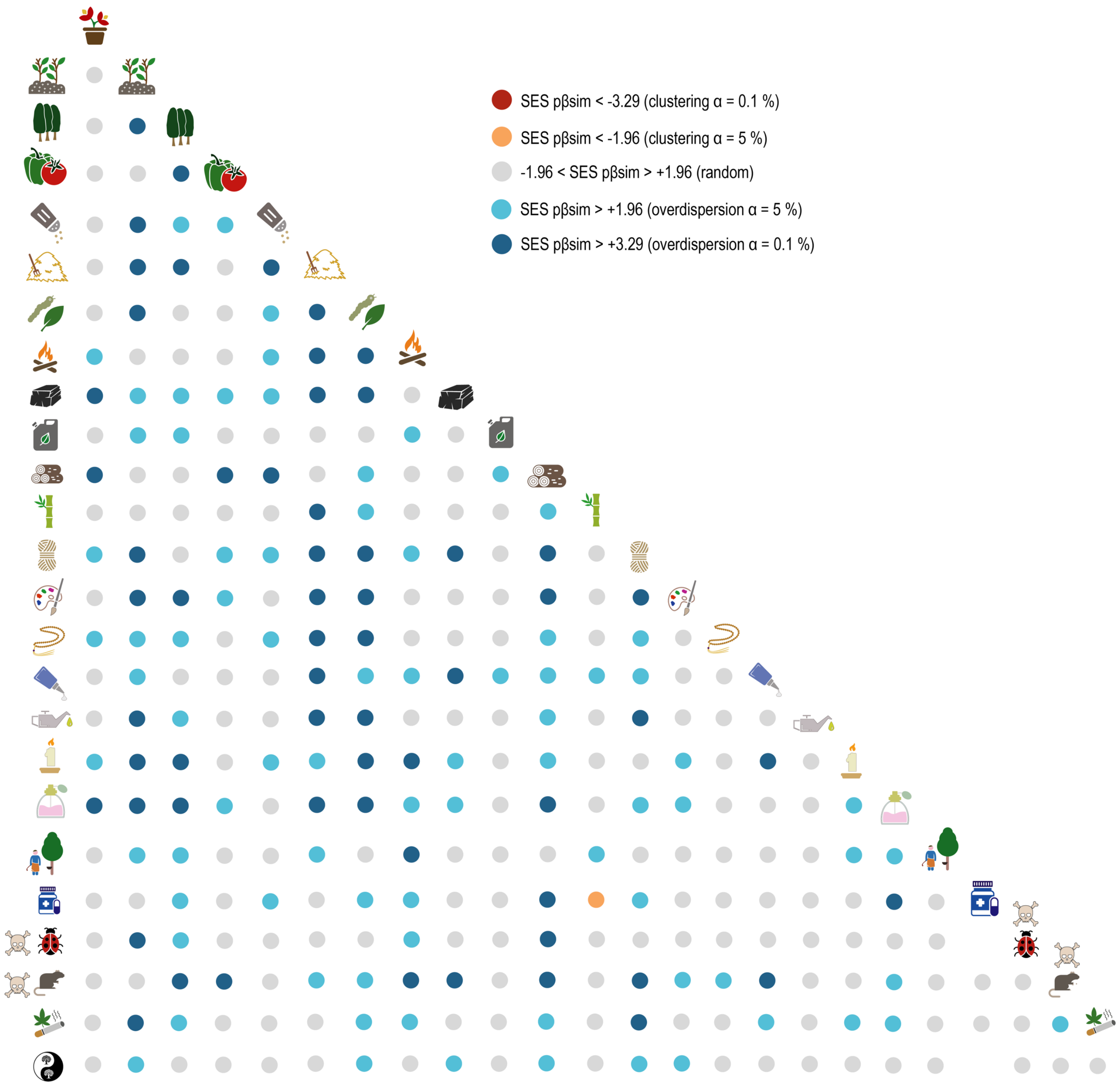

Supplementary Figure 5. Statistical significance (averaged Standardized Effect Size scores, two-tailed tests) of the "true" turnover component of phylogenetic beta diversity (SES $p\beta_{sim}$) between pairwise types of benefits for 5% and 0.1% nominal alpha (genus level and congeneric dataset). The exact averaged SES $p\beta_{sim}$ scores with 95% confidence intervals are provided in Supplementary Table 1, and they were considered significant only if 95% confidence intervals (representing phylogenetic uncertainty in SES score estimations) laid completely above (higher than expected) or below (lower than expected) the corresponding threshold (see legend).

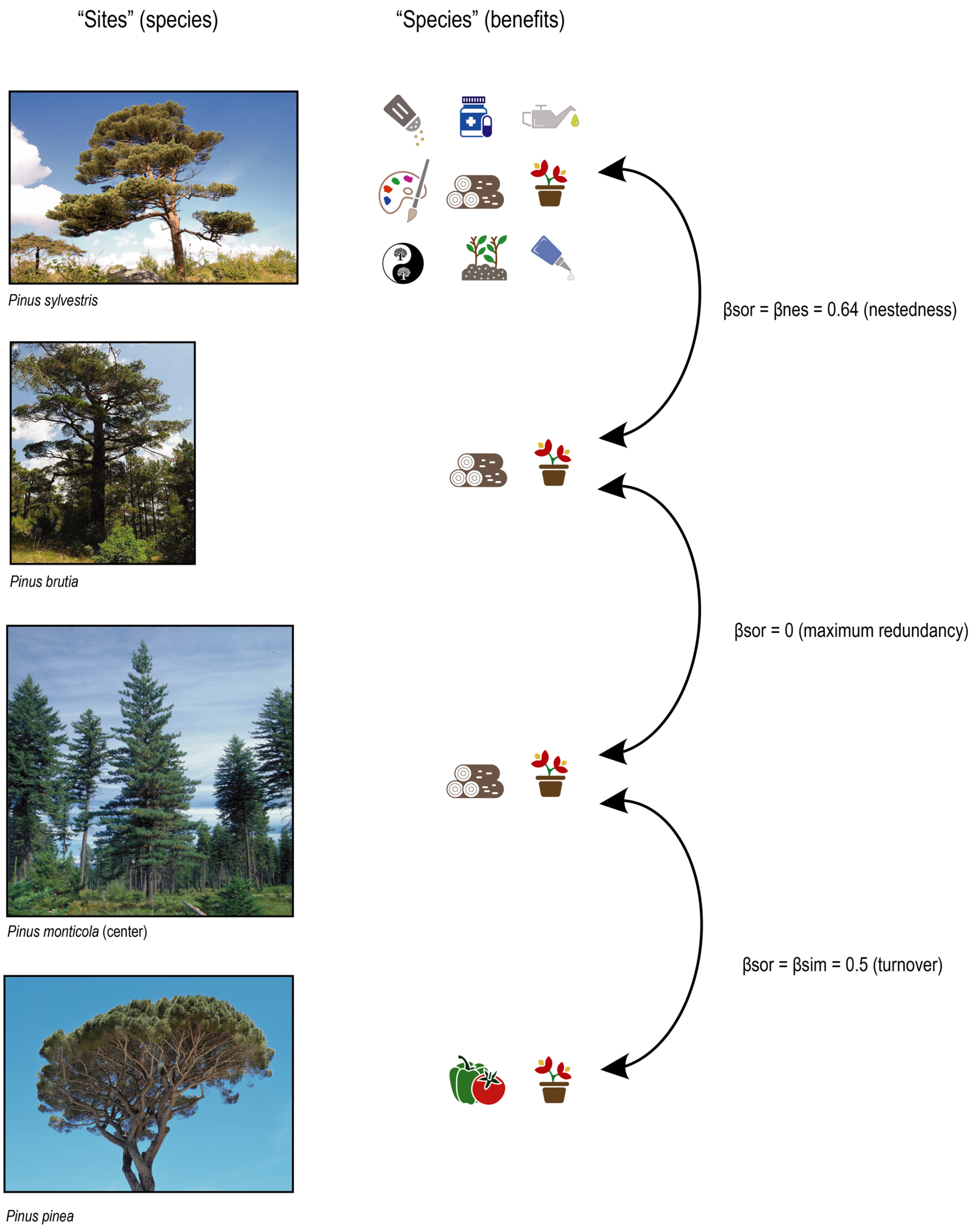

Supplementary Figure 6. Conceptual framework for the use of compositional beta diversity (β_{sor}) as an indicator of differential contribution of benefits among congeneric species (also applicable to confamiliars). The β_{sor} metric has been traditionally used to measure compositional dissimilarity between sites or communities (distance in taxonomic units). Here, however, I treated species as if they were “sites” and benefits as “species”. The “sites” *Pinus brutia* and *Pinus monticola* share the exact same “species” (benefits),

and thus β_{sor} between them is equal to zero (maximum redundancy in contributed types of benefits). In contrast, *Pinus brutia* and *Pinus pinea* only share one out of two contributed benefits, and thus there is some "true" benefit turnover between them (β_{sor} is entirely due to the β_{sim} component). Most congenerics in the dataset fit well two either of these schemes (i.e. they contributed very few and largely redundant types of benefits). However, I found that β_{nes} (compositional nestedness) was significantly high in many cases, revealing the signature of few species that stood out as multi-beneficial plants among their congenerics. *Pinus sylvestris* is one of such species, as in addition to the services already provided by the congenerics *Pinus brutia* and *Pinus monticola*, it contributed seven more types of benefits. Thus, β_{sor} between *Pinus sylvestris* and either *Pinus brutia* or *Pinus monticola* is entirely due to β_{nes} (compositional nestedness). Note that although here I described pairwise comparisons for didactical purposes, compositional dissimilarity in benefits was computed among all congenerics simultaneously (multi-site comparisons instead of pairwise) to elucidate the general pattern for each genus. Photos of *Pinus sylvestris* and *Pinus pinea* by Pxfuel (https://www.pxfuel.com). Photo of *Pinus brutia* by Javiermartin jc under CC-BY license (https://www.ecured.cu/Archivo:Pinus_brutia.jpg). Photo of *Pinus monticola* (center) by Chris Schnepf under CC-BY license (https://ca.wikipedia.org/wiki/Pinus_monticola).

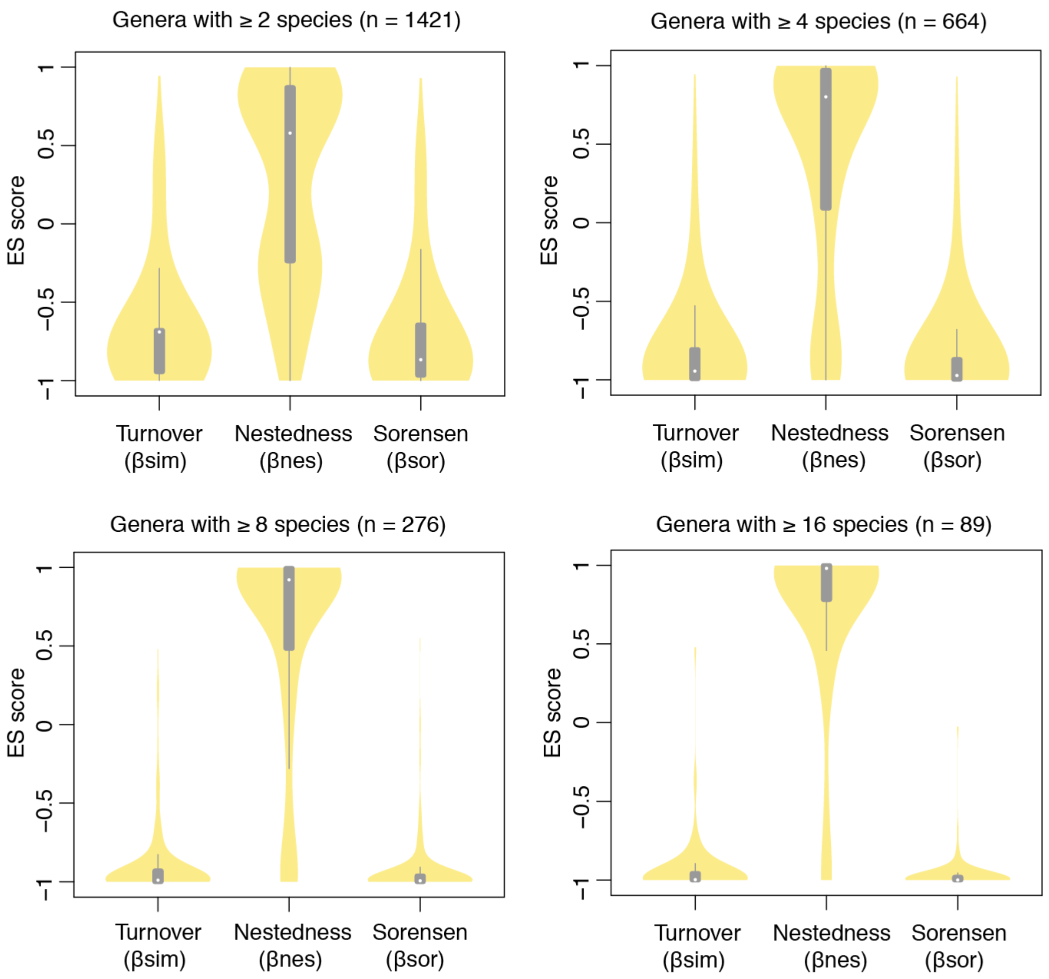

Supplementary Figure 7. Effect Size (ES) scores of multiple-site compositional turnover (β_{sim}) and nestedness (β_{nes}) in plant benefits among congenerics. Results are displayed for subsets of genera of increasing size (from top to bottom and left to right). ES scores for the overall dissimilarity among congenerics (β_{sor}) are also shown. The exact ES β_{sim}, β_{nes} and β_{sor} scores are provided in Supplementary Table 1.

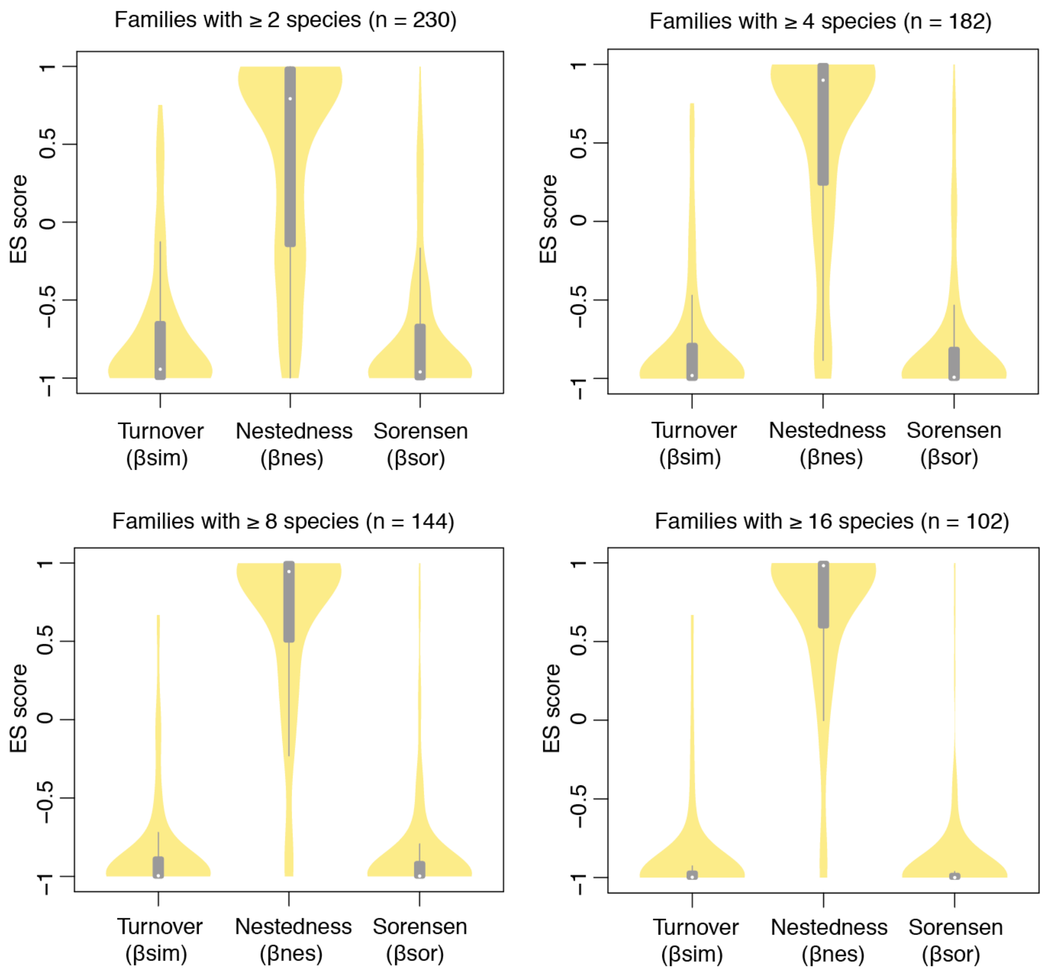

Supplementary Figure 8. Effect Size (ES) scores of multiple-site compositional turnover (β_{sim}) and nestedness (β_{nes}) in plant benefits among confamiliars. Results are displayed for subsets of families of increasing size (from top to bottom and left to right). ES scores for the overall dissimilarity among confamiliars (β_{sor}) are also shown. The exact ES β_{sim}, β_{nes} and β_{sor} scores are provided in Supplementary Table 1.

Supplementary Table 2. Averaged Standardized Effect Size (SES) scores with 95% confidence intervals of the median evolutionary distinctiveness of subsets of multi-beneficial plants (those contributing at least three, four, five, six, seven and eight types of benefits). SES scores relate to two different species pools, namely all seed plants and the beneficial species analyzed in the study. Averaged SES scores > 1.96 are significantly high for a 5% nominal alpha (two-tailed tests).

	Full dataset			Congeneric dataset		
Benefits	N	Seed plants pool	Beneficial species pool	N	Seed plants pool	Beneficial species pool
3+	1548	21.124 ± 0.104	6.827 ± 0.057	1328	15.501 ± 0.098	3.246 ± 0.052
4+	666	17.346 ± 0.095	7.023 ± 0.058	569	13.668 ± 0.090	4.462 ± 0.053
5+	302	15.091 ± 0.097	7.064 ± 0.066	257	11.702 ± 0.092	4.880 ± 0.064
6+	143	11.026 ± 0.091	5.372 ± 0.062	120	8.534 ± 0.085	3.521 ± 0.054
7+	73	9.134 ± 0.085	4.407 ± 0.055	57	7.102 ± 0.077	3.230 ± 0.050
8+	39	5.597 ± 0.068	2. 577 ± 0.046	29	4.189 ± 0.062	1.692 ± 0.040

Supplementary Table 3. Chi-square tests (one-tailed) on the difference between the observed number of benefits of each type contributed by multi-beneficial species (subsets of species with at least three, four and five benefits) and that expected given their representation in the pool of beneficial species (16.3%, 7% and 3.2%, respectively). The symbols "***", "**", "*" indicates the cases in which the null hypothesis cannot be rejected for a nominal alpha of 5%, 1% and 0.1%, respectively (one degree of freedom).

	3+ benefits (16.3% species pool)		4+ benefits (7% species pool)		5+ benefits (3.2% species pool)	
	% records contributed	χ^2	% records contributed	χ^2	% records contributed	χ^2
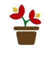	18.42	14.462	8.09	8.626*	3.71	4.559**
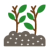	51.61	525.692	28.51	452.58	15.79	343.306
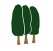	67.95	553.882	43.92	656.758	26.71	588.448
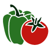	39.66	449.122	22.49	457.798	11.62	300.098
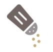	63.82	634.315	36.4	563.79	19.96	404.988
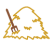	32.72	136.543	16.73	110.906	10.38	134.096
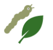	68.22	391.911	49.15	599.611	29.66	522.061
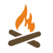	81.12	370.001	62.24	623.87	41.96	678.21
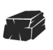	84.21	161.878	66.67	290.147	45.61	323.701
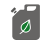	82.86	95.479	71.43	207.73	62.86	393.076
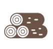	36.79	312.791	21.21	348.644	11.1	239.291
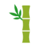	34.67	31.262	14.67	12.62	6	3.782***
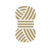	54.49	290.356	31.27	272.084	19.81	282.036
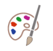	64.53	246.55	43.02	319.17	28.49	347.544
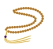	68.87	180.443	50	280.253	33.02	297.701
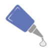	63.48	244.154	43.82	345.078	28.09	348.434
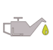	69.93	271.12	54.9	501.991	40.52	672.93
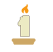	55.56	17.096	27.78	11.114	22.22	20.594
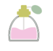	68.46	499.377	40.27	471.649	20.81	292.123
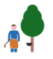	37.93	16.755	8.62	0.219***	6.9	2.537***
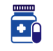	34.38	617.964	17.32	466.366	8.59	283.694
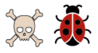	81.82	116.315	52.27	128.952	27.27	80.573
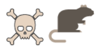	55.63	753.967	25.92	404.852	13.27	254.508
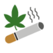	55.84	74.212	37.66	103.526	19.48	64.565
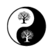	90.74	184.25	59.26	210.868	38.89	217.179

Supplementary Table 4. Chi-square tests (one-tailed) on the difference between the observed number of benefits of each type contributed by multi-beneficial species (subsets of species with at least six, seven and eight benefits) and that expected given their representation in the pool of beneficial species (1.5%, 0.8% and 0.4%, respectively). The symbols "***", "**", "*" indicates cases in which the null hypothesis cannot be rejected for a nominal alpha of 5%, 1% and 0.1%, respectively (one degree of freedom).

	6+ benefits (1.5% species pool)		7+ benefits (0.8% species pool)		8+ benefits (0.4% species pool)	
	% records contributed	χ^2	% records contributed	χ^2	% records contributed	χ^2
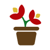	1.8%	3.067***	1.01%	3.929**	0.56%	2.607***
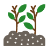	8.77%	240.697	4.97%	157.67	2.78%	93.647
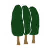	15.43%	435.285	10.09%	381.975	6.82%	338.597
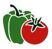	6%	179.462	3.3%	111.51	1.8%	62.875
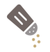	12.28%	352.735	6.14%	171.735	3.29%	92.326
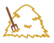	5.37%	81.688	2.32%	25.768	1.1%	9.499*
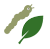	21.19%	608.846	11.44%	350.689	6.78%	233.784
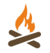	25.87%	565.551	18.88%	611.989	13.29%	578.881
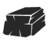	36.84%	473.978	24.56%	420.915	19.3%	496.471
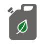	51.43%	580.87	34.29%	512.873	17.14%	239.247
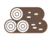	5.72%	142.754	3.15%	89.289	1.82%	58.838
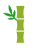	2%	0.248***	1.33%	0.628***	0%	0.614***
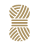	9.91%	151.93	6.81%	153.912	3.1%	56.905
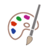	18.6%	334.969	11.63%	264.632	7.56%	214.575
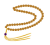	25.47%	405.489	16.98%	363.469	11.32%	308.08
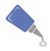	17.98%	321.698	11.8%	282.496	7.3%	206.514
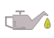	29.41%	793.509	19.61%	708.377	12.42%	538.642
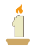	22.22%	51.453	11.11%	25.121	11.11%	50.324
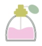	8.72%	103.511	4.7%	60.068	2.01%	18.713
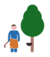	1.72%	0.019***	0%	0.445***	0%	0.238***
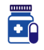	4.28%	157.354	2.16%	77.125	1.18%	43.93
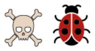	20.45%	105.229	9.09%	39.765	4.55%	18.374
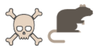	6.19%	115.978	3.79%	94.462	2.28%	67.237
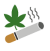	9.09%	29.526	5.19%	19.692	5.19%	43.043
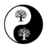	22.22%	154.359	12.96%	104.762	5.56%	34.909

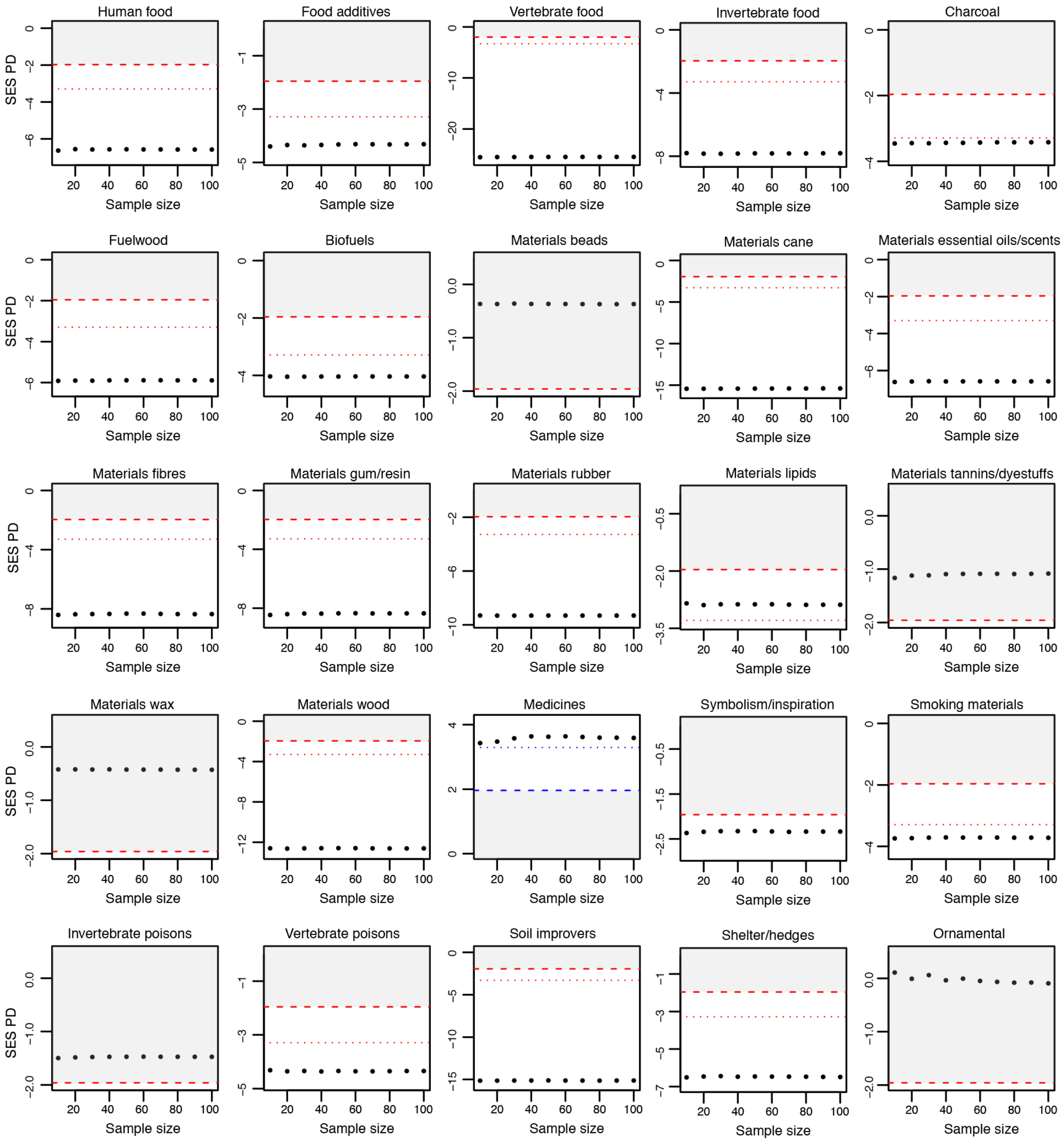

Supplementary Figure 9. Sensitivity of phylogenetic analyses to species-level phylogenetic uncertainty. The scatter plots display averaged SES PD scores for the benefits (full dataset) using an increasing number of species-level trees that were derived from the same genus-level phylogeny. The thick and thin dotted lines represent the significance thresholds for a nominal alpha of 5% and 0.1%, respectively (in red and blue for the left and right sides of the distribution, respectively), and the shaded areas span the range of non-significant values for a 5% alpha. Note that the statistical significance of PD was insensitive to the number of species-level trees used to average SES scores.

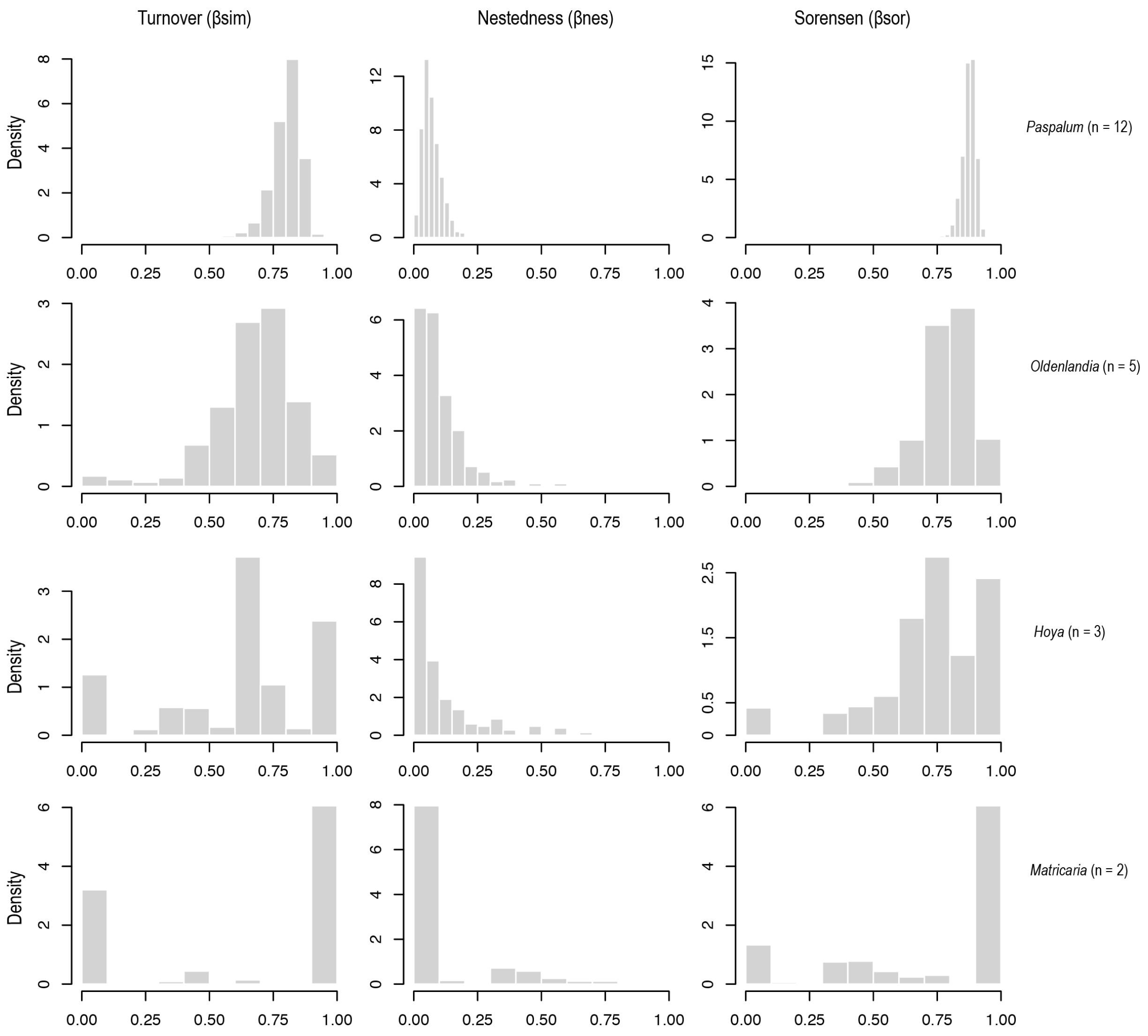

Supplementary Figure 10. Null distributions of compositional dissimilarity in benefits among congenerics (β_{sor}) and its "true" turnover (β_{sim}) and nestedness components (β_{nes}). The data correspond to four randomly picked genera of different size that were sampled in the percentile 90th (*Paspalum*, 12 species), 70th (*Oldenlandia*, 5 species), 50th (*Hoya*, 3 species) and 30th (*Hoya*, 2 species) of the distribution of species richness per genus, respectively. Note that the distributions were odd and did not fit normality, particularly for small-sized genera.
